# Core procedural skills in the MD curriculum: what students need to learn

**DOI:** 10.3389/fmed.2024.1469781

**Published:** 2024-10-23

**Authors:** Thuy Minh Ha, Hai Ngoc Truong, Zarrin Seema Siddiqui

**Affiliations:** ^1^Adjunct Faculty, College of Health Sciences, VinUniversity, Hanoi, Vietnam; ^2^Internal Medicine Department, Vinmec Healthcare System, Hanoi, Vietnam; ^3^Adjunct Professor in Medical Education, Health Services Academy, Islamabad, Pakistan

**Keywords:** curriculum development, clinical practice, medical education, procedure skills, Vietnam

## Abstract

The medical education system in Vietnam is currently undergoing a significant transformation with the support of the Ministry of Education and Training (MOET) and the Ministry of Health (MOH). Alongside this shift, the emergence of new medical schools, including VinUniversity, reflects a growing enrollment trend in undergraduate medical programs (UME). During the curriculum development phase, a crucial gap in core procedural skills among medical graduates surfaced. In response, the Medical Doctor (MD) Education team launched a project aimed at establishing a consensus-based list of essential procedural skills and identifying optimal clinical placements for skill acquisition. A cross-sectional survey, based on this list, was distributed among physicians and residents in the VINMEC Healthcare system, asking them to rate skills using the Likert scale and specify suitable clinical placements or rotations for acquisition. With 207 respondents, a consensus list of 38 core procedural skills was generated and integrated into the curriculum. This adaptable list holds promise for adoption by medical schools nationwide, fostering standardization and enhancement of medical education quality.

## Introduction

The landscape of medical education in Vietnam is currently experiencing a significant transformation with the support of the Ministry of Education and Training (MOET) and the Ministry of Health (MOH) (1). Concurrently, a wave of new medical institutions is surfacing, with a steady surge in student enrollment annually. Presently, Vietnam boasts more than 30 medical universities, each admitting thousands of students yearly (1) University curricula follow a national framework for Medical Doctor (MD) program but vary from institution to institution (2). The absence of a standardized national licensing examination poses a challenge in evaluating the caliber of training and ensuring alignment with societal demands (3).

The medical education system in Vietnam is structured as a six-year undergraduate program leading to the Medical Doctor (MD) degree (2). Students begin their medical education immediately after completing high school. The first two to three years are dedicated to basic sciences and preclinical subjects, laying the foundation for medical knowledge. The subsequent three years focus on clinical education, during which students rotate through core medical disciplines, including Internal Medicine, Surgery, Pediatrics, Obstetrics and Gynecology, as well as various other specialties such as Neurology, Psychiatry, and Dermatology (2). Upon completing the six-year program, graduates are considered general practitioners; however, they are not yet licensed to practice independently. To specialize in a particular field, graduates must pass a competitive national examination known as the ‘Residency Entrance Exam’, which allows them to choose their specialty (1). Residency training in Vietnam typically lasts three years, depending on the chosen specialty. Alternatively, graduates may opt to work in hospitals for a required period before applying for Specialist Training I and II in their chosen specialty, which ranges from three to five years (1).

VinUniversity is a newly established institution, offering undergraduate medical education (UME) with the first cohort graduating in 2026. The MD Education Unit at the College of Health Sciences is responsible for planning, implementation and evaluation of the MD Program. As part of the curriculum development, the unit engaged stakeholders including community, clinicians and recent graduates. The purpose of the engagement is primarily to identify expectations which serve as the cornerstone for curriculum development and resource preparation (4). Within the clinical training setting, determination of the procedural skills essential for undergraduate medical students, along with identifying the optimal clinical environments for their acquisition, became imperative. A quick review of existing documents and guidelines revealed that while a generic curriculum framework was endorsed by MOET in 2012, it lacked specific learning objectives for the clinical years (5). In 2015, further guidelines were issued outlining the competencies expected of medical graduates; however, these too fell short of specifying the procedural skills required for the training program (6). Previous studies on curriculum reform across eight public medical universities in Vietnam between 1999 and 2006 highlighted a consensus among stakeholders regarding the knowledge, attitudes, and skills expected of medical graduates (7). However, the generalizability of these findings to other medical schools remains unproven (8). The existing curricula from various medical schools in Vietnam identifies required skills and varied approaches to teaching and learning those skills (7). Existing curricula across various medical schools in Vietnam indicate varied approaches to teaching and learning these skills (9).

Given this context, the present study aims to investigate the essential procedural skills required in the core curriculum. The specific objectives of this study were (i) to identify the essential procedural skills required of a medical graduate in Vietnam and (ii) where in the clinical setting these skills can be better learned by medical students.

## Methods

A cross-sectional study was designed with a questionnaire survey which was sent through email to all medical doctors working at VINMEC chain of hospitals across Vietnam. The survey comprises three sections.

- Section one: This section listed all procedural skills identified through document scanning and review of relevant literature. Participants were asked to evaluate each skill on a 5-point Likert scale, where 1 = Not at all important, 2 = Less likely important, 3 = Maybe important, 4 = Essential, and 5 = Very essential.
- Section two: Participants were required to indicate the most suitable clinical setting for acquiring each procedural skill. This section aimed to identify where within the clinical setting these skills could be most effectively learned.
- Section three: This section collected demographic data which includes gender, current designation and discipline with the year respondent graduated as medical doctor.

The survey was designed in both English and Vietnamese to ensure accessibility for all participants.

As part of the validation of the survey, a group of physicians from VINMEC hospitals were invited to participate in a detailed briefing session. During the session, the research team thoroughly explained the purpose of the study and provided participants with a paper-based version of the survey. Participants were asked to complete the survey and offer feedback, particularly noting any ambiguities, difficulties, or issues with the Vietnamese translation. Twenty participants attended the session, and their feedback was instrumental in refining the survey items, ensuring that all questions were clearly understood and accurately reflected the intended content. Based on their input, the survey was revised and finalized to include thirty-eight core procedural skills. The response options in Section one were rephrased to include the response options: “not at all,” “less likely,” “good to know,” “essential,” and “very essential.” Sections two and three remained unchanged, with the addition of a free text option for further comments and feedback.

An invitation to participate in the online survey was emailed to all physicians (*n* = 350) employed across the five hospitals within the VINMEC healthcare system. The email provided detailed information about the study and emphasized that participation was entirely voluntary. Participants were informed of the consent process before beginning the survey, and it was made clear that no identifiable data, such as names or hospital affiliations, would be recorded to ensure confidentiality. The survey was distributed over a period from March to June 2020.

## Results

Two hundred and seven responses were recorded, representing about 60% of the target population. Table 1 presents the demographic characteristics of the respondents. The demographic analysis shows that 56.5% of participants were male and 43.5% female. Most respondents (37.9%) had 10–20 years of experience, with 26.7% having over 20 years. Specialists made up 74.4% of the sample, residents 16.4%, general practitioners 6.8, and 2.4% were educators not actively involved in clinical practice.

**Table 1:** Respondent demographics.

		Frequency	Percentage
		(*N* = 207)	%
Gender	Male	117	56.5
	Female	90	43.5
Year since graduation	<5 years	34	16.5
	5–10 years	39	18.9
	10–20 years	78	37.9
	>20 years	56	26.7
Current status	General Practitioner	14	6.8
	Resident	34	16.4
	Specialist	154	74.4
	Educator (not in active practice)	05	2.4

For the survey items, participants were asked to rate each procedural skill using a 5-point Likert scale, with scores ranging from 1 (Not at all important) to 5 (Very essential). The mean score for each skill was calculated and converted into a percentage to facilitate the categorization of skills based on their perceived importance. The following thresholds were used to interpret the results:

- Essential: Skills with a mean score above 70%.
- Good to Know: Skills with a mean score between 50 and 70%.
- Less Likely: Skills with a mean score between 30 and 49%.
- Not at All: Skills with a mean score below 30%.

The results, as presented in Table 2, highlight a prioritized list of core procedural skills across different categories.

**Table 2:** Core procedural skills categorized.

No	Categories of core procedural skills
	**Essential**
1	First Aid Management of patient
2	Cardiopulmonary resuscitation (CPR)
3	Venipuncture
4	Anterior nasal packing
5	Arterial puncture
6	External splinting
7	Suturing
8	Digital block
9	Oxygen therapy
10	Blood and blood component transfusion
11	Endotracheal intubation
12	Aerosol bronchodilator therapy
13	Thoracentesis
14	Normal labor
15	Stomal care
16	Central line
17	Breathing exercise
18	Insert speculum
19	Incision and drainage of skin and sub cutaneous tissues
20	Postural drainage
21	Bedside ultrasound
	**Good to know**
22	Reset dislocated joints
23	Vaginal packing
24	Aspiration of skin, subcutaneous tissues and bursa
25	Pap smear
26	Strengthen and stretching exercise
27	Episiotomy
28	Removal of foreign body from vagina in adult
	**Less Likely**
29	Excision of benign tumor
30	Apply massage and acupressure
31	Perform stool examination for worm eggs using direct microscopy
32	Biopsy of skin
33	Polypectomy
34	Insertion and removal of intra uterine device
35	Cervical biopsy
	**Not at all**
36	Insert acupuncture needles
37	Amniotomy
38	Marsupialization of Bartholin’s cyst

The “Essential” category includes the top three skills: First Aid Management, Cardiopulmonary Resuscitation (CPR), and Venipuncture underscores their priority in medical training. Skills categorized as “good to know” such as resetting dislocated joints and performing a Pap smear, are important but context-dependent, often necessary in specific clinical scenarios or patient populations. The “less likely” category includes procedures like the excision of benign tumors and the application of massage and acupressure, which are perceived as less frequently required in general practice. Finally, the “not at all” category includes skills such as acupuncture needle insertion and Marsupialization of Bartholin’s cyst, reflecting their limited relevance to the core requirement for medical graduates.

The findings of the optimal clinical settings for learning essential procedural skills, as detailed in Appendix 1, identifies core clinical rotations as the most recommended environments. Specifically, Internal Medicine, Surgery, Obstetrics and Gynecology, Pediatrics, and Emergency/ICU were highlighted as ideal placements for acquiring a diverse range of skills. Each of these rotations was noted to offer opportunities to acquire approximately five essential procedural skills, underscoring their critical contribution in medical training.

In the open-ended comments, participants identified skills which are not procedural skills, for example communication skills. Additionally, there were suggestions to enhance First Aid courses to better reflect contemporary medical needs, indicating a broader interest in adapting the curriculum to meet evolving healthcare challenges.

## Discussion

Feedback and insights from physicians with their experience in both clinical practice and medical education can help in defining the curriculum which is aligned with the current demands of the field. As the result of this study, a catalog of procedural skills is compiled and integrated into the curriculum, offering a resource that can also benefit other medical institutions across the country. As the clinical environment is intense and students must move from one rotation to another within a span from four to eight weeks, the MD Education team has integrated the procedural skills among medical students right from the year one using simulation center where students are able to practice the skills within safe environment in addition to an intense transition to clinical practice block (4 weeks) before year 4.

The study highlights the importance of First Aid, Cardiopulmonary Resuscitation, and Intravenous skills, as the top three skills which are universally recognized as critical components of medical education. Conversely, skills like Acupuncture Needle Insertion, Amniotomy, and Marsupialization of Bartholin’s Cyst received low priority. Traditional medicine plays an important role in the Vietnamese society and though acupuncture may not be perceived as a necessary skill for all medical graduates, it may be part of electives offered. Skills like Amniotomy and Marsupialization of Bartholin’s Cyst may be more appropriately taught at the postgraduate level, given their specialized application in specific medical contexts.

The study also highlights the significance of aligning clinical rotations with the procedural skills being taught. For instance, while specialized rotations such as Obstetrics and Gynecology or Pediatrics are crucial for acquiring specific skills, many essential skills, such as Arterial Puncture, Blood and blood component transfusion, Oxygen therapy, Postural drainage, and Venipuncture, can be effectively learned across multiple disciplines, including Internal Medicine, Surgery, and Emergency/ICU. Remarkably, the five most essential skills identified align with those that participants believe can be learned in any department including Arterial puncture; Blood and blood component transfusion; Oxygen therapy; Postural drainage; Venipuncture.

The study’s findings have provided our institution with an inventory of procedural skills crucial for medical education. With the identified procedural skills as a foundation, assessment methods will be refined to better align with student evaluations, ensuring a curriculum that is both pragmatic and tailored to undergraduate needs.

### Limitations and future directions

The study contains some limitations that should be acquired. Primarily, the study focused on procedural skills within core clinical disciplines, such as Internal Medicine, Surgery, Pediatrics, Obstetrics and Gynecology, and Emergency Medicine. Although these areas are vital for immediate clinical practice, the exclusion of other specialties such as psychiatry and neurology limit the comprehensiveness of the identified skill set. Future research should aim to include these and other critical specialties to ensure a more holistic approach to medical education.

Another significant limitation is the absence of a detailed subgroup analysis, which could have provided valuable insights into how different participant groups, such as specialist physicians and residents, prioritize procedural skills. Specialist physicians, for instance, might emphasize more advanced skills due to their greater clinical responsibilities, whereas residents may focus on fundamental skills essential in the early stages of their careers (10). Additionally, the study did not examine the correlation between the duration of post-specialization work and the prioritization of skills. Understanding how clinical focus evolves with experience could offer important guidance for curriculum development.

The generalizability of the findings is also limited by the study’s focus on a specific group of medical professionals within the VINMEC Healthcare system. To enhance the applicability of the results, future studies should involve a broader range of institutions and healthcare settings. This would allow for a more comprehensive understanding of essential procedural skills across diverse medical education contexts.

In the future, the next phase of this research will focus on determining the level of competence required for each identified skill and the number of attempts necessary to achieve proficiency. This will help to refine assessment methods and ensure that student evaluations are aligned with the practical demands of clinical practice.

## Conclusion

By identifying expectations and involving potential employers and stakeholders from the onset, the program is expected to be more engaging, relevant and effective. This study offers a reference point for other medical schools seeking to structure their curricula and enhance the quality of instruction to better meet societal needs. Following graduation, medical students are presented with opportunities for further education and clinical practice at the postgraduate level, as well as lifelong learning endeavors. The consensus reached on essential procedural skills offers institutions a strategic tool to optimize their resources, ensuring that medical education is both efficient and impactful.

## Data Availability

The original contributions presented in the study are included in the article/supplementary materials, further inquiries can be directed to the corresponding author.

## Appendix

**Table A1:** Clinical rotation where skills can be learned.

No.	Procedural skills	Suggested clinical rotations
1	Aerosol bronchodilator therapy	Internal Medicine and sub-specialties; Pediatrics; Emergency/ICU
2	Arterial nasal packing	Internal Medicine and sub-specialties; Surgery and sub-specialties; Emergency/ICU
3	Breathing exercise	Internal Medicine and sub-specialties; Surgery and sub-specialties; Pediatrics; Other specialty (Rehabilitation, physical therapy)
4	Endotracheal intubation	Internal Medicine and sub-specialties; Surgery and sub-specialties; Pediatrics; Emergency/ICU
5	Bedside ultrasound	Internal Medicine and sub-specialties; Surgery and sub-specialties; Obstetrics and Gynecology; Pediatrics; Emergency/ICU; Other specialty (Radiology)
6	Strengthen and stretching exercise	Internal Medicine and sub-specialties; Surgery and sub-specialties; Other specialty (Rehabilitation, physical therapy)
7	Thoracentesis	Internal Medicine and sub-specialties; Surgery and sub-specialties; Emergency/ICU
8	Digital block	Internal Medicine and sub-specialties; Emergency/ICU
9	Perform stool examination for worm eggs using direct microscopy	Internal Medicine and sub-specialties; Other specialty (Pathology)
10	Aspiration of skin, subcutaneous tissues, and bursa	Surgery and sub-specialties; Emergency/ICU
11	Biopsy of skin	Surgery and sub-specialties
12	Excision of benign tumor	Surgery and sub-specialties
13	External splinting	Surgery and sub-specialties; Emergency/ICU
14	First Aid Management of patient	Surgery and sub-specialties; Emergency/ICU
15	Polypectomy	Surgery and sub-specialties; Obstetrics and Gynecology
16	Stomal care	Surgery and sub-specialties
17	Suturing	Surgery and sub-specialties; Emergency/ICU
18	Reset dislocated joints	Surgery and sub-specialties
19	Incision and drainage of skin and subcutaneous tissues	Surgery and sub-specialties
20	Amniotomy	Obstetrics and Gynecology
21	Cervical biopsy	Obstetrics and Gynecology
22	Episiotomy	Obstetrics and Gynecology
23	Insertion and removal of intra uterine device	Obstetrics and Gynecology
24	Marsupialization of Bartholin’s cyst	Obstetrics and Gynecology
25	Pap smear	Obstetrics and Gynecology
26	Normal labor	Obstetrics and Gynecology
27	Removal of foreign body from vagina in adult	Obstetrics and Gynecology
28	Vaginal packing	Obstetrics and Gynecology
29	Insert speculum	Obstetrics and Gynecology
30	CPR	Emergency/ICU
31	Central line	Emergency/ICU
32	Insert acupuncture needles	Other specialty (Traditional Medicine)
33	Apply massage and acupressure	Other specialty (Traditional Medicine)
34	Arterial puncture	Can be taught in any specialty
35	Blood and blood component transfusion	Can be taught in any specialty
36	Oxygen therapy	Can be taught in any specialty
37	Postural drainage	Can be taught in any specialty
38	Venipuncture	Can be taught in any specialty
